# Force Field Parameterization for the Description of the Interactions between Hydroxypropyl-β-Cyclodextrin and Proteins

**DOI:** 10.1021/acs.jpcb.1c04033

**Published:** 2021-07-02

**Authors:** Andrea Arsiccio, Marcello Rospiccio, Joan-Emma Shea, Roberto Pisano

**Affiliations:** † Department of Chemistry and Biochemistry, University of California, Santa Barbara, California 93106, United States; ‡ Molecular Engineering Laboratory, Department of Applied Science and Technology, Politecnico di Torino, 24 corso Duca degli Abruzzi, Torino 10129, Italy; § Department of Physics, University of California, Santa Barbara, California 93106, United States

## Abstract

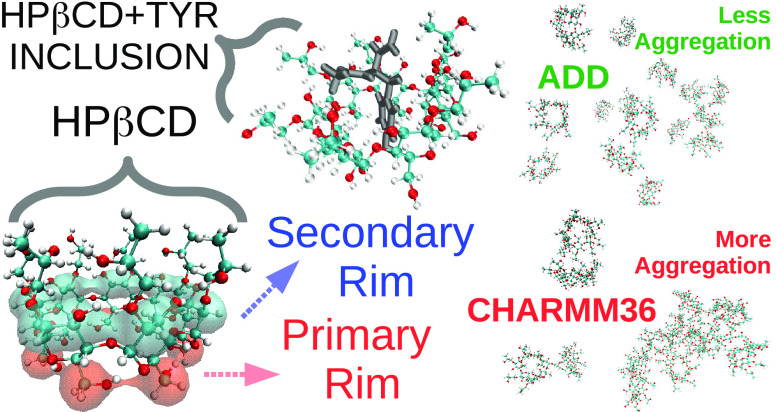

Cyclodextrins are cyclic oligosaccharides, widely used as drug carriers, solubilizers, and excipients. Among cyclodextrins, the functionalized derivative known as hydroxypropyl-β-cyclodextrin (HPβCD) offers several advantages due to its unique structural features. Its optimal use in pharmaceutical and medical applications would benefit from a molecular-level understanding of its behavior, as can be offered by molecular dynamics simulations. Here, we propose a set of parameters for all-atom simulations of HPβCD, based on the ADD force field for sugars developed in our group, and compare it to the original CHARMM36 description. Using Kirkwood–Buff integrals of binary HPβCD–water mixtures as target experimental data, we show that the ADD-based description results in a considerably improved prediction of HPβCD self-association and interaction with water. We then use the new set of parameters to characterize the behavior of HPβCD toward the different amino acids. We observe pronounced interactions of HPβCD with both polar and nonpolar moieties, with a special preference for the aromatic rings of tyrosine, phenylalanine, and tryptophan. Interestingly, our simulations further highlight a preferential orientation of HPβCD’s hydrophobic cavity toward the backbone atoms of amino acids, which, coupled with a favorable interaction of HPβCD with the peptide backbone, suggest a propensity for HPβCD to denature proteins.

## Introduction

Cyclodextrins (CDs) are cyclic oligosaccharides containing 6 (αCD), 7 (βCD), or 8 (γCD) α-glucopyranose monomers. CDs possess a unique torus-like-shaped structure and are characterized by the presence of a lipophilic cavity and a hydrophilic outer surface. The internal core is surrounded by two rims (primary rim, formed by C6 atoms of the glucopyranose subunits, and secondary rim, consisting of the C2 and C3 atoms).

CDs count several applications as excipients, drug carriers, solubilizers, and adsorption enhancers.^1−3^ They can increase the solubility and bioavailability of hydrophobic drugs by including them within their lipophilic cavity. Because of their distinct features, CDs are present in many marketed drugs, and their field of application is supposed to grow further in the next few years. For instance, CDs have been widely used in formulations for oral, parenteral, nasal, pulmonary, and skin delivery of drugs,^4−6^ and there is widespread interest in their use for delivery to the brain.^4,7,8^

Among CDs, βCD has arisen particular interest due to its structural characteristics. For instance, the cavity size of βCD allows the inclusion of aromatic amino acids, such as Phe, Tyr, His, and Trp, and this mechanism is supposed to be at the basis of the effective prevention of protein aggregation observed for this CD and its derivatives.^1,9−12^ However, βCD is poorly soluble in water (the solubility is only 16 mM at 25 °C), and this makes it unsuitable for parenteral formulations. This problem can nevertheless be mitigated by the addition of hydrophilic derivatizations to the glucopyranose subunit, for instance, by substituting some hydroxyl groups with other moieties. Among the possible derivatizations, hydroxypropyl groups may be linked to the glucopyranose monomers, eventually obtaining the so-called hydroxypropyl-β-cyclodextrin (HPβCD). HPβCD is more soluble than its unfunctionalized counterpart (>300 mM at ambient temperature) and displays amphiphilic properties. For instance, it was found to be surface-active, as such mitigating surface-induced aggregation of proteins.^13−16^

HPβCD is approved as an excipient in parenteral formulations and was authorized by both FDA (in 2010) and EMA (in 2013) for the treatment of the Niemann–Pick type C (NPC) disease. NPC disease is a progressive neurodegenerative disorder characterized by the intracellular accumulation of cholesterol.^17^ The effectiveness of HPβCD against NPC is related to its action as a cholesterol-scavenging compound, capable of transporting cholesterol away from cell bodies.^18,19^ An elevated level of cholesterol is also a risk factor for Alzheimer’s disease, suggesting that HPβCD may also prove beneficial for its treatment. However, observations on this point are conflicting.^20^

The pharmaceutical and medical applications of HPβCD would benefit from a better characterization of its behavior at the molecular level, as could be provided by molecular dynamics simulations. For this purpose, a force field capable of describing with good accuracy HPβCD–water and HPβCD–protein interactions is needed. We recently developed a force field for sugars and polyols,^21^ named ADD, and showed that it can correctly describe sugar–protein and sugar–water interactions, as well as self-association of sugars. We developed the new parameters in combination with the CHARMM36m^22^ force field for proteins, but good compatibility was also observed with other extensively used force fields, such as the AMBER 99SB-ILDN^23^ and the OPLS-AA^24^ force fields. The ADD parameters represent, therefore, a very promising candidate for the description of the α-glucopyranose subunit of cyclodextrins.

**Figure 1:**
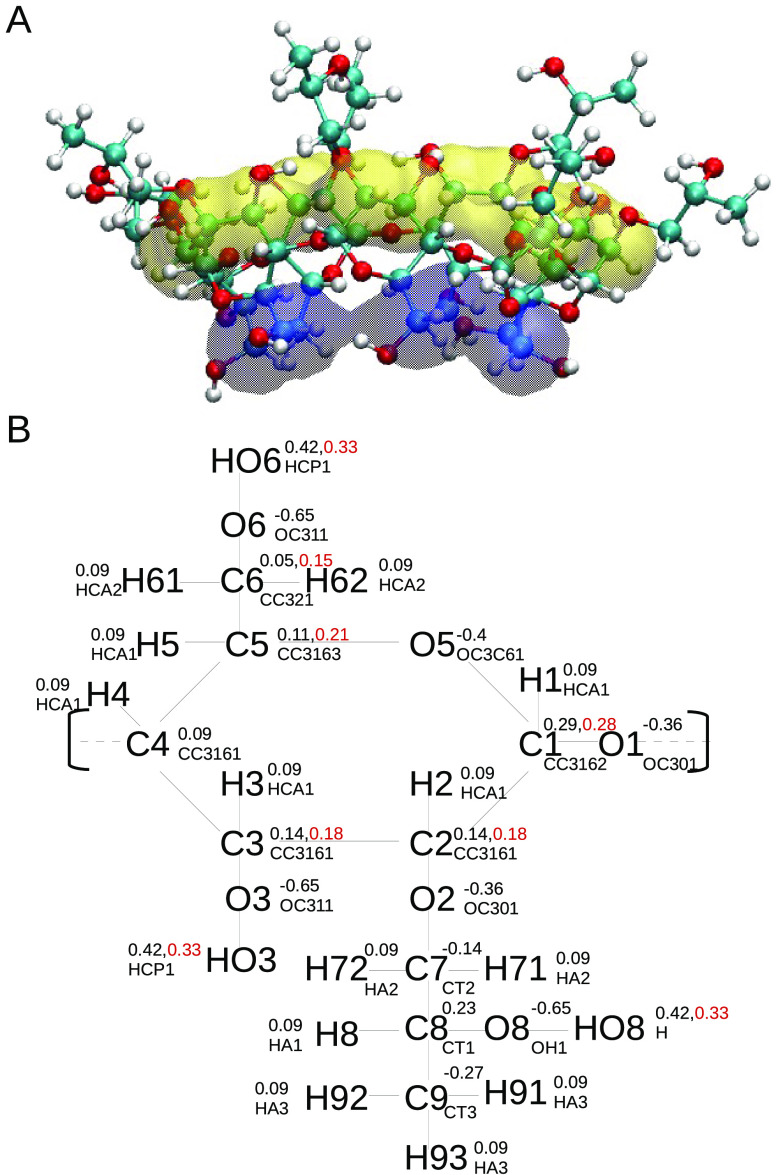
(A) Ball-and-stick representation of 2-HPβCD. The primary and secondary rims are highlighted in blue and yellow, respectively. (B) Schematic representation of the 2-HPβCD subunit. The atom types and partial charges used for the simulations are also shown. When different charges are employed for the CHARMM36 and ADD force fields, they are shown with different colors (black for the original CHARMM36 and red for ADD).

We will here test the application of the ADD force field to CDs, comparing the output to previous force fields^25^ and available experimental data. In particular, the Kirkwood–Buff integrals^26−29^ will be used as target experimental data for validation because they were found to be an excellent benchmark for force field development.^21,30−33^ We will focus our attention on HPβCD because of its unique properties and numerous applications and because of the extensive experimental characterization available for this functionalized CD. Different possible forms of HPβCD exist, depending on the degree of substitution and position of the derivatization, and we here study the form where the hydroxypropyl group is linked to the O2 atom of the glucose unit (2-HPβCD) and fully substituted for all of the seven residues. A ball-and-stick representation of the 2-HPβCD variant studied in this work is shown in Figure 1A, where the primary and secondary rims have been highlighted.

In the following, we will first validate the ADD description for 2-HPβCD and show how it outperforms previous force fields. We will then study the interaction of 2-HPβCD with the 20 naturally occurring amino acid side chains and the peptide backbone to gain an understanding of protein–HPβCD systems at the molecular level.

## Materials and Methods

### Theoretical Background

The ADD force field for 2-HPβCD will be validated against experimental quantities related to the Kirkwood–Buff theory of solutions.^26^ In this context, a central role is played by the Kirkwood–Buff integrals (KBIs), *G*_*ij*_, which are used to describe the solvation behavior of component *j* around a reference particle *i*
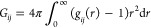
1*g*_*ij*_(*r*) is the radial distribution function (RDF), which describes the variations in component *j* density as a function of the distance *r* from component *i*. A value of *G*_*ij*_ < 1 indicates exclusion, while *G*_*ij*_ > 1 indicates accumulation of component *j* around the reference *i*.

From now on, we will refer to a system containing water (component 1), HPβCD (component 3), and, optionally, also an amino acid or peptide (component 2).

For a binary water–HPβCD mixture (no component 2), the KBIs are related to the composition of the solution (molar density of water ρ_1_ and HPβCD ρ_3_), to the chemical potential of HPβCD μ_3_, and to the partial molar volumes of water (*V*_1_) and HPβCD (*V*_3_) as follows^28^
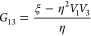
2
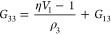
3
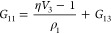
4where
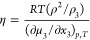
5
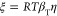
6
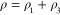
7where *R* is the universal gas constant, *T* and *p* are temperature and pressure, respectively, *x*_*i*_ are mole fractions, ρ is the total molar density of the solution, and β_T_ is the isothermal compressibility.

Knowing *G*_13_, *G*_33_, and *G*_13_, it is then possible to compute other properties. For instance, the derivative of the mole fraction scale activity coefficient *f*_3_ with the mole fraction *x*_3_ can be evaluated as follows^28^
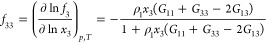
8In a ternary solution containing also an amino acid or peptide (component 2), the KBIs can provide interesting information about the HPβCD–peptide interactions. For instance, the difference γ = *G*_23_ – *G*_12_ quantifies the preferential interaction or exclusion of component 3 from component 2. Specifically, a negative value of γ indicates preferential exclusion and vice versa.

### Simulation Details

Molecular dynamics simulations were carried out using Gromacs 2018.6.^34^ Two different force fields were compared for 2-HPβCD. In the first case, the original CHARMM36 force field was used for the glucopyranose subunits,^25^ while the hydroxypropyl derivatization was modeled with parameters obtained by analogy, as provided by the CHARMM general force field (CGenFF) program.^35^ In the second case, charges were adjusted to comply with the ADD force field for carbohydrates.^21^ In the ADD force field, the partial charges of the hydrogen atom in the hydroxyl group are modified compared to the original CHARMM36 force field (0.33 for H in the ADD description, compared to 0.42 in the original CHARMM36). Because of this, also the other charges need to be modified to preserve the overall neutrality, and the atom types and charges used for the simulations are shown in Figure 1B.

An additional difference of the ADD description compared to the original CHARMM36 resides in the Lennard-Jones parameter ε of the O and H atoms of the hydroxyl group, as shown in Table 1. Specifically, the original CHARMM36 description uses two different combinations of ε values for hydroxyl groups within the glucopyranose subunit or in the hydroxypropyl derivatization. In contrast, the ADD force field employs a single set of ε values for all hydroxyl groups. No difference exists between the two force fields for what concerns the Lennard-Jones parameters σ.

**Table 1:** Lennard-Jones Parameters ε for the Hydroxyl Groups of HPβCD

force field	ε(O)_glucopyranose_ (kJ/mol)	ε(H)_glucopyranose_ (kJ/mol)	ε(O)_hydroxypropyl_ (kJ/mol)	ε(H)_hydroxypropyl_ (kJ/mol)
original CHARMM36	0.804	0.192	0.636	0.192
ADD	0.450	0.120	0.450	0.120

For all simulations, the CHARMM TIP3P water model was used,^36^ while, whenever present, amino acids were described according to the CHARMM36m force field.^22^ A scheme of all simulations performed, with the corresponding box size, temperature, and duration, is listed in Table 2.

**Table 2:** List of the Simulations Performed in This Work

sim. type (#)	solute (component 2)	HPβCD conc. (mol/L) (component 3)	box size (nm)	*T* (K)	duration (ns)
1		0.061–0.123–0.186	8 × 8 × 8	298	60
2	capped amino acids	0.050	8 × 8 × 8	300	60
3	NAG_*x*_A	0.050	8 × 8 × 8	300	100

In sim. type 1, three different concentrations of HPβCD were considered in the range of 61–186 mM. These simulations were carried out with the objective to extract selected properties of binary water–HPβCD solutions (density, Kirkwood–Buff integrals, derivative of activity coefficient) to be then compared with experimental data. A temperature of 298 K was selected for these simulations to allow a direct comparison with the experimental data used for validation.

For sim. type 2, all of the 20 naturally occurring amino acids, in their capped form (i.e., acetylated N-terminus and amidated C-terminus), were simulated in 50 mM HPβCD. Also, the N-acetyl glycinamide series (NAG_*x*_A) was simulated (sim. type 3 in Table 2). NAG_*x*_A corresponds to a series of molecules with a varying number *x* of glycine residues linked by a peptide bond and whose termini are blocked by an acetyl (N-terminus) and an amide (C-terminus) moiety. The number of internal glycine units has been varied from 1 (NAG_1_A) to 6 (NAG_6_A). For simulations 2 and 3, the box was cubic with an ≈8 nm side length and included 25 amino acid/NAG_*x*_A molecules. For charged residues, Na^+^ or Cl^–^ ions were added to reach neutrality.

In all cases, the cutoff radius for both Coulombic (calculated using the PME method^37^) and Lennard-Jones interactions was set to 1.2 nm, and periodic boundary conditions were used. Each box was first energy-minimized with the steepest descent algorithm and then equilibrated for 1 ns at 1 bar and 298 K (sim. 1) or 300 K (sim. 2–3) in the NPT ensemble, using Berendsen pressure (3 ps relaxation time) and temperature (0.5 ps relaxation time) coupling.^38^ The simulations were then run at the same temperature used for equilibration and at 1 bar in the NPT ensemble, controlling temperature and pressure with the Nosé–Hoover thermostat^39,40^ (0.5 ps relaxation time) and Parrinello–Rahman barostat^41^ (3 ps relaxation time), respectively. A 2 fs time step was used, and configurations were saved every 2 ps. The Lincs algorithm was employed for constraining all bonds,^42^ while the SETTLE algorithm was used to keep the water molecules rigid.^43^ The last 50 ns (for sim. type 1) or 40 ns (for sim. types 2 and 3) were used for the analyses.

### Analyses of Simulation Results

#### Kirkwood–Buff Integrals

The Kirkwood–Buff integrals were calculated by taking the average of the running KBIs
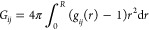
9at values of *R* where convergence is reached. To correct for finite size effects, the correction suggested in ref (44) was applied. Briefly, corrected radial distribution functions were computed as
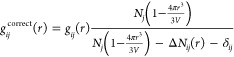
10where *N*_*j*_ is the number of particles of type *j* in the system, *V* is the system volume, Δ*N*_*ij*_(*r*) is the excess number of particles *j* within a sphere of radius *r* around *i*, and δ_*ij*_ is the Kronecker delta.

#### Amino Acid Inclusion within the HPβCD Cavity

An amino acid was deemed to be included within the HPβCD cavity when it was closer than 0.5 nm to the center of mass of HPβCD. The 0.5 nm cutoff was selected so as to guarantee that only amino acids really included within the cavity, and not simply close to it, were counted. The number of CDs involved in inclusions was counted for each frame, averaged over the total number of frames and subsequently normalized by the total number of cyclodextrins in the system to allow a direct comparison of the different simulations.

#### Hydrogen Bonding

The number of amino acid–HPβCD hydrogen bonds was computed. The χ-parameter was evaluated as follows
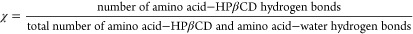
11To determine the presence of a hydrogen bond, a geometrical criterion was used, requiring that the distance between the donor and acceptor was less than 0.35 nm and that the angle formed between the hydrogen atom and the line joining the center of masses of donor and acceptor was smaller than 30°.

## Results and Discussion

### Validation of the ADD Force Field for HPβCD

**Figure 2:**
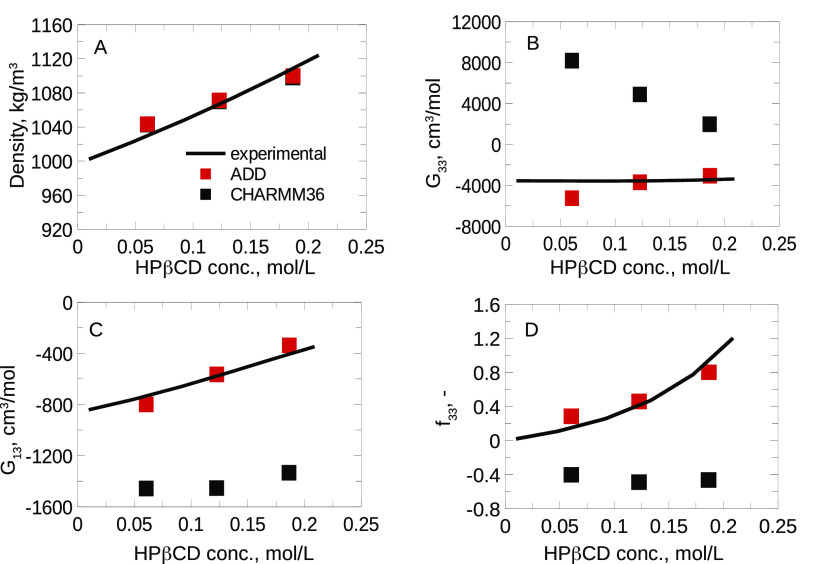
Comparison between experimental (solid black line) and simulation results (black squares, original CHARMM36; red squares, ADD force field). (A) Solution density, (B) HPβCD–HPβCD Kirkwood–Buff integral *G*_33_, (C) HPβCD–water Kirkwood–Buff integral *G*_13_, and (D) derivative of the mole fraction scale activity coefficient with respect to mole fraction.

The first objective of the present work was to compare the original CHARMM36 and the ADD force fields, using target experimental data as reference. The simulations type 1 listed in Table 2 were performed for this purpose. The experimental data considered in this work were the solution mass density, the Kirkwood–Buff integrals *G*_33_ and *G*_13_, and the derivative of the mole fraction scale activity coefficient with respect to the mole fraction. The solution density and the partial molar volume of HPβCD (*V*_3_ that comes into play in eqs 2 and 4) were obtained from ref (45). Due to the low concentration of HPβCD considered in this work (the maximum value we simulated was only 0.186 mol/L), we assumed the partial molar volume of water (*V*_1_ = 18.07 cm^3^/mol) and the isothermal compressibility (β_T_ = 4.52 × 10^–10^ Pa^–1^) to be approximately constant. The molal fraction scale activity coefficient *f*_3,*m*_ of 2-HPβCD was obtained from ref (46) and converted to the mole fraction scale using the relation^47^*f*_3,*m*_ = *x*_3_*f*_3_. From *f*_3_, it was then possible to compute *f*_33_ = (∂ ln *f*_3_/∂ ln *x*_3_)_*p*,*T*_ and (∂μ_3_/∂*x*_3_)_*p*,*T*_ = *RT*[(∂ ln *f*_3_/∂*x*_3_) + 1/*x*_3_]. The values of (∂μ_3_/∂*x*_3_)_*p*,*T*_, *V*_1_, *V*_3_, and β_T_ were then substituted into eqs 5 and 6 to compute η and ξ and, eventually, the KBIs through eqs 2–4. The experimental properties computed in this way were compared to the simulation results, as shown in Figure 2.

It is evident that the ADD parameters result in a considerably improved description of binary water–HPβCD mixtures. The original CHARMM36 force field fails in predicting the *G*_33_ and *G*_13_ Kirkwood–Buff integrals (Figure 2B,C) and also the derivative of the activity coefficient (Figure 2D). For instance, the root-mean-square error between experimental and simulated data of *f*_33_ is 1.01 for the original CHARMM36 and decreases to only 0.12 when the ADD parameters are used. The two force fields overlap only for the description of density (Figure 2A). It follows that the ADD force field should be preferred over the original CHARMM36 for the description of HPβCD–water mixtures.

### HPβCD Interacts with Polar and Apolar Side Chains but Is Excluded from Charged Moieties

Simulations type 2 in Table 2 were used to study the interaction of HPβCD with the different amino acids. In particular, the values of γ = *G*_23_ – *G*_12_ were extracted from the simulations.

We were particularly interested in studying the interaction of HPβCD with the different side chains and the protein backbone separately. For this purpose, we followed the approach proposed by Auton et al.^48,49^ and already employed in our previous work.^21^ Briefly, we assumed the existence of additivity for the γ values and computed the side-chain contribution γ_*i*_^sc^ by subtracting the KBI for glycine γ_gly_ to the KBI of the specific amino acid *i* being considered γ_*i*_
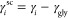
12Capped amino acids were used for this purpose. As shown in ref (21), the side-chain contribution γ_*i*_^sc^ is not influenced by the terminal capping conditions (i.e., zwitterionic vs capped form). This is also in line with previous observations by Nozaki and Tanford,^50^ who showed that the interaction of the side chain of a branched organic compound with the solvent was approximately independent of the interaction of the backbone to which the side chain was attached.

**Figure 3:**
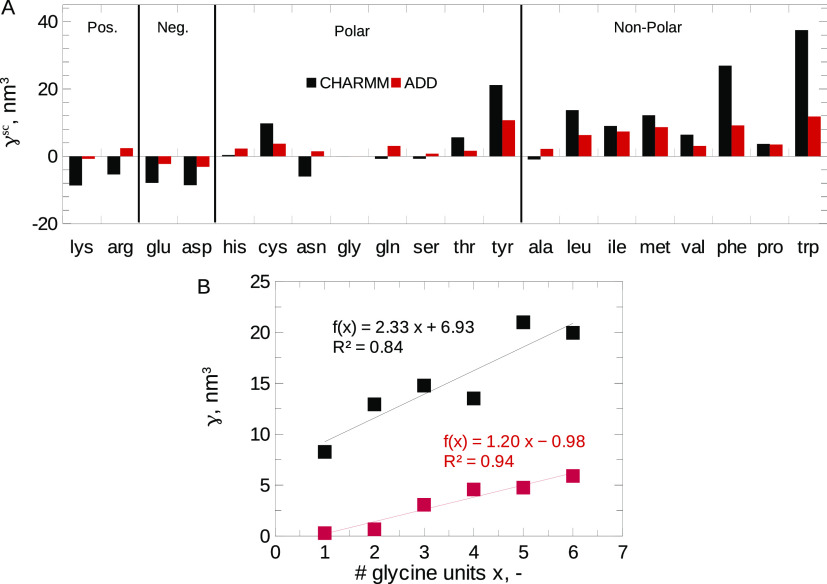
(A) KB integrals γ^sc^ = γ_*i*_ – γ_gly_ for the amino acid side chains. The amino acids are divided into groups depending on their side-chain properties (positively or negatively charged, polar and nonpolar moving from left to right). Capped amino acids were used for the simulations. (B) KB integrals (γ = *G*_23_ – *G*_12_) for the N-acetyl glycinamide series NAG_*x*_A as a function of the number of internal glycine units *x*. Black, original CHARMM36; red, ADD force field.

The values of γ_*i*_^sc^ obtained in this way are displayed in Figure 3A. On average, HPβCD was found to be excluded (negative γ_*i*_^sc^), or only marginally interacting, with charged side chains. This was true for both the original CHARMM36 and ADD force fields and is in line with what observed in Arsiccio et al.^21^ for sucrose and sorbitol. However, this may be a limitation of the force field, as a mismatch between simulations and experiments was noted for charged moieties.^21^

The interaction with polar side chains was mostly favorable, with the only exception of asparagine, glutamine, and serine, for which a very small or negative value of γ_*i*_^sc^ was measured.

The interaction was favorable, and quite intense, also for most apolar side chains, with the only exclusion of alanine, that showed a value of γ_*i*_^sc^ close to zero. The ability of HPβCD to interact with both polar and apolar side chains is not surprising considering the amphiphilic nature of this excipient, which was already the subject of both experimental and computational investigation.^13,14,16^

The interaction of HPβCD with aromatic side chains (especially tryptophan, phenylalanine, and tyrosine) was particularly pronounced. The preferential interaction of HPβCD with aromatic groups has been observed experimentally and is well documented in the literature.^9−12^ The entity of the interaction was substantially different for the two force fields, with the ADD parameters resulting in less pronounced interaction with polar/apolar groups and reduced exclusion from charged side chains. However, it is interesting and important to note that the trend was very similar for both the original CHARMM36 and the ADD force fields, with an almost perfect agreement in the predicted sign of the γ_*i*_^sc^ values.

### HPβCD–Backbone Interaction: Evidence of a Denaturing Behavior

Having characterized the HPβCD–side-chain interactions, we now set to study the interaction with the backbone. The backbone contribution γ^bb^ can be computed according to the constant increment method^51^ applied to the NAG_*x*_A series (sim. type 3 in Table 2)
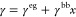
13where the value of γ for the entire NAG_*x*_A is decomposed into the end group contribution (γ^eg^) and the backbone contribution (γ^bb^) multiplied by the number of internal glycine units *x*. γ^bb^ can therefore be obtained by fitting a straight line through the γ values as a function of the number of internal glycine units, as shown in Figure 3B.

The γ^bb^ values obtained are positive for both the original CHARMM36 and the ADD force fields (2.33 and 1.20 nm^3^, with good coefficients of determination *R*^2^ values of 0.84 and 0.94, respectively). This points to a favorable interaction of HPβCD with the protein backbone. According to Auton et al.,^48,52^ this is indicative of a denaturing character of HPβCD. The denaturing nature of this cyclodextrin is also in line with previous experimental investigations for immunoglobulin G formulations,^53^ where increasing HPβCD concentrations were found to reduce the protein melting temperature. As previously noted for the side-chain contributions already, the original CHARMM36 and ADD force fields differ in the entity of the predicted γ^bb^ but not in the sign of the interaction.

### HPβCD Preferentially Orients with Its Secondary Rim toward the Amino Acid Backbone

We previously mentioned that HPβCD is an amphiphilic molecule, with two rims (Figure 1A) characterized by different properties. Hence, we analyzed the orientation of HPβCD toward the different amino acids.

**Figure 4:**
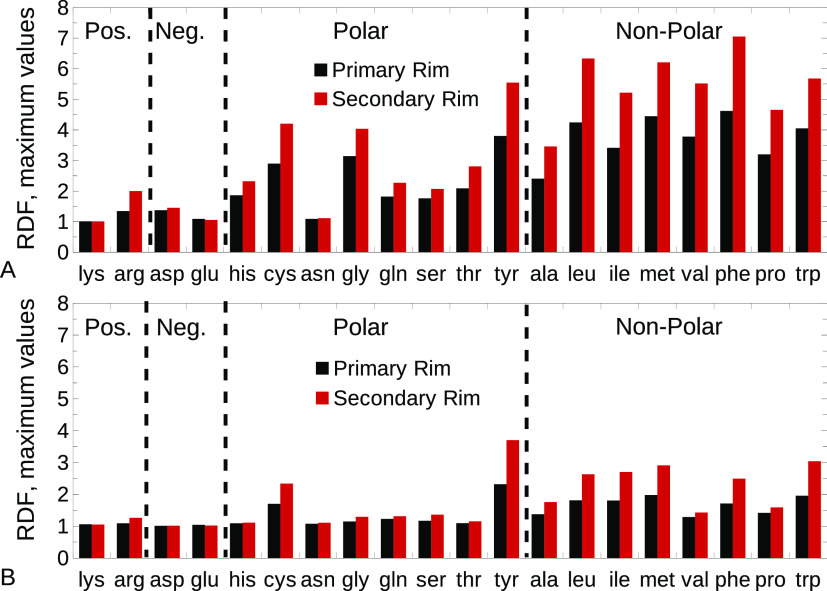
Maximum values of the RDFs between the primary and secondary rims of HPβCD and the center of mass of the amino acids for the original CHARMM36 (A) and ADD (B) force fields. Black, primary rim; red, secondary rim.

For this purpose, we computed the radial distribution functions (RDFs) for the atoms of both rims, with respect to the center of mass of the amino acids, and extracted the maximum value of these RDFs (Figure 4). We can observe that, in general, HPβCD mostly oriented with its secondary rim toward the amino acids. The preferential orientation of HPβCD was not very pronounced only for charged amino acids, likely because of the marginal interactions between the CD and the charged moieties as already evidenced in Figure 3A. Again, the overall trend was similar for both the CHARMM36 and ADD force fields, with the ADD description resulting in weaker interactions.

**Figure 5:**
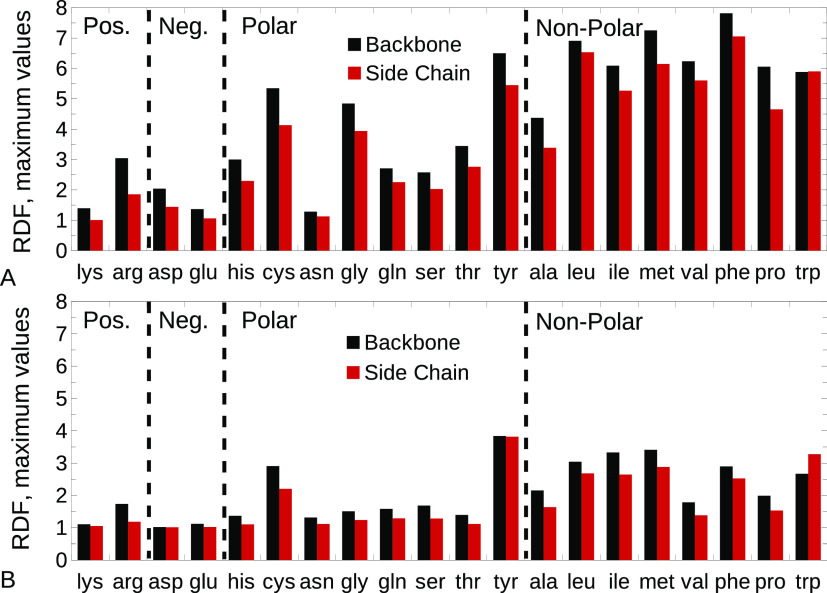
Maximum values of the RDFs between the backbone or side-chain atoms of the different amino acids, and the center of mass of the HPβCD secondary rim, for the original CHARMM36 (A) or ADD (B) force fields. Black, backbone; red, side chain.

After having established that HPβCD preferentially orients with its secondary rim toward the amino acids, we verified whether it was closer to the side chain or the peptide backbone. For this purpose, we calculated the RDFs between the backbone or side-chain atoms of the different amino acids and the center of mass of the HPβCD secondary rim. The maximum values of the RDFs obtained in this way are shown in Figure 5. The trend observed suggests that interactions preferentially occur between the hydrophobic cavity (secondary rim) of the CD and the peptide backbone, in line with the favorable HPβCD–backbone interaction evidenced in Figure 3B. Tryptophan constituted, however, a noticeable exception. In this case, HPβCD strongly interacted with the aromatic ring of the tryptophan side chain, in line with experimental works suggesting a preferential affinity of CDs towards aromatic groups.^9−12^

**Figure 6:**
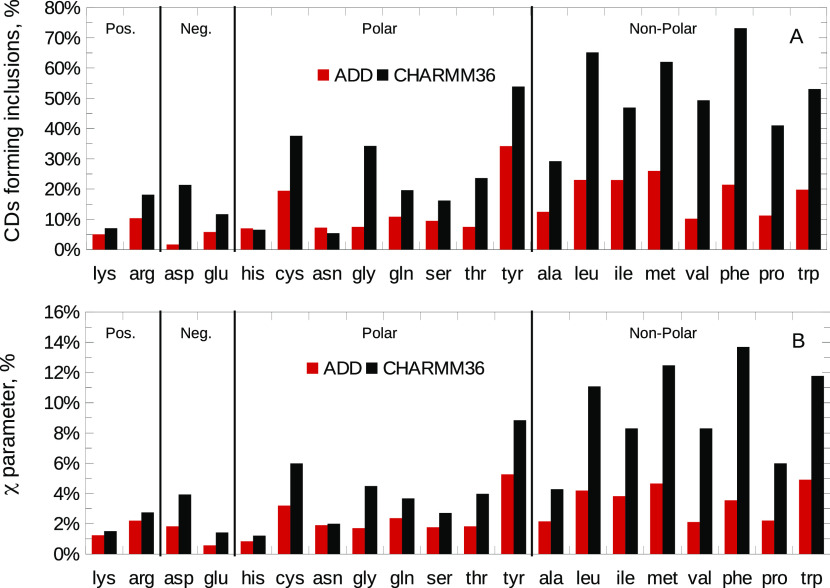
(A) Percentage of HPβCD molecules including amino acids during the equilibrated trajectory. (B) χ parameter, i.e., percentage of hydrogen bonds established between HPβCD and the amino acids. Black, original CHARMM36; red, ADD force field.

HPβCD may also form inclusions with amino acids. We calculated the percentage of HPβCD molecules that, on average, included amino acids within their cavity during the equilibrated trajectories (Figure 6A). We found that the occurrence of inclusions was in agreement with the previously discussed trends of γ^sc^ (Figure 3A) and maximum values of the RDFs (Figures 4 and 5). Again, inclusion was negligible for charged residues and more pronounced for polar and apolar ones, especially for the aromatic residues tyrosine, phenylalanine, and tryptophan. The original CHARMM36 and ADD force fields displayed the same overall trend, although the degree of interaction was lower with the ADD description. These same conclusions could be drawn looking at the formation of hydrogen bonds (χ parameter; eq 11), as displayed in Figure 6B. This figure suggests that hydrogen bonding contributes to HPβCD–amino acid interactions.

## Conclusions

HPβCD is emerging as an important agent for drug delivery, as its unique structural characteristics make it particularly well suited to increase the solubility and bioavailability of hydrophobic drugs through encapsulation. In this work, we have developed a new force field for HPβCD that accurately describes its physical properties. The original CHARMM36 force field was used as the starting point for this investigation, and we have exposed its limitations in reproducing experimental data of binary water–HPβCD mixtures. We then proposed and validated a new force field based on the ADD description of sugars,^21^ which accurately reproduces HPβCD–HPβCD and HPβCD–water interactions.

We have applied the new force field to the characterization of HPβCD–amino acid interactions, quantified in terms of Kirkwood–Buff integrals (KBIs), and compared our results to the original CHARMM36 description. We have observed that HPβCD only marginally interacts with charged side chains, while it exhibits strong affinities toward most of the apolar moieties, especially the aromatic ones. For polar side chains, strong interaction was noted with the aromatic ring of tyrosine and the thiol side chain of cysteine. The trends observed for the CHARMM36 and ADD force fields were similar, but CHARMM36 predicted considerably stronger interactions. The poor interaction between charged groups and HPβCD, observed with both CHARMM36 and ADD, may, however, be a limitation of the force fields, as a mismatch between simulations and experiments has previously been observed for charged moieties in ref (21).

Both the CHARMM36 and ADD force fields evidenced a favorable interaction of HPβCD with the peptide backbone, which may be indicative of its denaturing behavior. In line with this, we further found that HPβCD mainly oriented with its secondary rim, i.e., the hydrophobic cavity, toward the peptide backbone. However, the aromatic amino acid tryptophan strongly interacted with HPβCD also through its side chain.

As cyclodextrins are known for their ability to form complexes with small molecules, we evaluated the degree of amino acid inclusion inside the HPβCD’s cavity. We observed that the tendency to form inclusion complexes with different amino acids reflected the chemical affinity highlighted by our KBIs. Inclusion was also accompanied by an increased number of hydrogen bonds between the cyclodextrins and the amino acids.

We believe that the improved description of HPβCD provided in this paper will prove to be useful for the investigation of this promising excipient, enabling the use of computational techniques, such as molecular dynamics, to further clarify its properties. A better understanding of HPβCD–protein mixtures would be of great interest in the pharmaceutical, biotechnological, and medical fields. In this respect, the characterization of HPβCD–amino acid interactions described here constitutes the first step toward unraveling this molecule’s behavior toward proteins.
